# mSphere of Influence: Understanding atopic disease through the dynamics of skin-microbe interactions

**DOI:** 10.1128/msphere.00797-25

**Published:** 2026-07-10

**Authors:** Flavia G. Costa

**Affiliations:** 1 Department of Immunology and Microbiology, University of Colorado—Anschutz Medical Campus 129263, Aurora, Colorado, USA; University of Nebraska Medical Center College of Medicine, Omaha, Nebraska, USA

**Keywords:** atopic dermatitis, bacteriology, host-microbe interactions

## Abstract

In this mSphere of Influence article, Flavia G. Costa explores dynamics of host-microbe interactions on the skin through three papers that have shaped her perspectives in this field. She first discusses a publication by Radhouani et al., which showed how *S. aureus* skin colonization promotes lung inflammation through eosinophil reprogramming. She then highlights the potential protective benefits of understudied skin commensals as described by Myles et al., finally concluding with a recent study demonstrating how byproducts from industrialization can disrupt these beneficial interactions as described by Zeldin et al. This body of work has shaped her approach toward understanding the microbial dynamics that contribute to the rise in incidence of atopic diseases.

## COMMENTARY

Throughout my training as a bacteriologist, I have been driven to understand the mechanisms underlying bacterial survival in their respective environments, an interest that was recently reflected in my efforts to understand *Staphylococcus aureus* physiology during human skin colonization. Currently, I am applying my research interests toward elucidating the pathways that contribute to the protective properties of understudied skin commensals. My current research is driven by the observation that changes in lifestyle, hygiene, and environmental exposures in the past century correlate with an increased incidence of several allergic and inflammatory diseases (1, 2). Additionally, industrialization correlates with a reduction in human microbiome diversity and disappearance of commensal species (3–5). Although causation cannot be determined from these data, together they highlight a need to better understand the role that commensals and environmental exposures play in immune signaling.

The potential consequence of reduced microbial diversity in human health is best exemplified in the case of atopic dermatitis (AD), where inflammation and development of skin lesions correlate with reduced cutaneous bacterial diversity and the increased incidence of the opportunistic pathogen *Staphylococcus aureus* (6). The development of AD or epicutaneous sensitization is also associated with increased risk for development of other atopic diseases, such as asthma and food allergies, in a phenomenon often referred to as the atopic march (7). Here, I will discuss three papers that have had a significant impact on my perspective on the mechanisms that drive AD and how our skin commensals are an important intermediary in our interactions with our environment.

## MICROORGANISMS CAN BE DRIVERS OF ALLERGIC INFLAMMATION

In a paper authored by Radhouani et al. titled “Eosinophil innate immune memory after bacterial skin infection promotes allergic lung inflammation,” the authors demonstrated that *S. aureus* plays a role in antigen sensitization and activation of the inflammatory responses observed in patients with AD and asthma (8). Using C57Bl/6 mice, this work showed that application of *S. aureus* to tape-stripped skin amplified and prolonged the inflammatory response, resulting in higher expression of type II inflammation-associated cytokines compared to the uninoculated tape-stripped control. These experiments are interesting, as they demonstrate a potentially causative role for *S. aureus* colonization in activating type II inflammation. These phenotypes were further exacerbated by subsequent exposure to house dust mite extract, a common household allergen, and this resulted in enhanced allergen sensitization of the lungs. These researchers observed that *S. aureus* skin colonization increased eosinophil recruitment to the skin and altered transcriptional programming of bone marrow-derived eosinophils, demonstrating the widespread effect of *S. aureus* skin colonization on the host’s immune system. This paper provides compelling evidence that *S. aureus* skin colonization can play a role in sensitization of other organs to allergens, supporting an earlier observation that topical application of staphylococcal superantigen enterotoxin B enhances allergic lung inflammation (9). This paper excited me because it suggests that bolstering commensal-mediated colonization resistance of *S. aureus* could not only reduce *S. aureus* colonization and infection risk, but also potentially have widespread protective effects on allergic inflammation. This work inspired me to take a closer look at skin commensals with properties that could contribute to *S. aureus* colonization resistance.

## SOME MICROORGANISMS CAN COUNTERACT ALLERGIC INFLAMMATION

If pathogens like *S. aureus* can promote allergic inflammation, are there commensals that are able to counteract this response? In work titled “Therapeutic responses to *Roseomonas mucosa* in atopic dermatitis may involve lipid-mediated TNF-related epithelial repair” published by Myles et al., the authors sought to characterize the mechanism behind the treatment of AD by topical application of *R. mucosa*, a gram-negative skin commensal (10). They found that application of *R. mucosa* at various dosing intervals and concentrations was associated with improved clinical outcomes for AD patients and reduced *S. aureus* burden on the skin, and that these improvements persisted for up to 8 months after cessation of treatment. Treatment was also associated with a reduction in cytokines TNFα and IL-13 in patients’ serum, and the authors found that a combination of TLR5 activation and production of ceramide-containing glycerophospholipids by *R. mucosa* as likely mechanisms producing this anti-inflammatory outcome. Interestingly, the authors noted that these effects varied across isolates, and, notably, none of the *R. mucosa* strains isolated from AD patients had this positive effect. This intraspecies variability in host responses to commensal microorganisms highlights the microbial diversity influencing these interactions and supports previous observations of intraspecies diversity in CoNS like *Staphylococcus epidermidis* (11). In summary, this work inspired me to investigate how other microorganisms present on the skin interact with host cells and counteract inflammatory signaling.

## OUR CHANGING ENVIRONMENT INFLUENCES HOST-MICROBE INTERACTIONS

As a follow-up to the previous work, Dr. Myles’ group published a manuscript titled “Exposure to isocyanates predicts atopic dermatitis prevalence and disrupts therapeutic pathways in commensal bacteria” led by Jordan Zeldin. This work showed that diisocyanate release from industrial activity is strongly correlated with pediatric clinical presentation of AD (12). The authors found that these pollutants inhibited growth of *R. mucosa* by disrupting nitrogen fixation and inhibiting production of alpha-linolenic acid. They went on to show that the presence of this class of pollutants disrupted the beneficial effects of *R. mucosa* in the previously established mouse model for AD and altered metabolism of CoNS species. This work not only connected skin microbial metabolism to host response, but it is also the first publication demonstrating a specific mechanism for how modern environmental exposures are changing skin commensal metabolism. As my primary interest is in bacterial metabolism and physiology, I found this work to be both personally interesting and impactful to our understanding of how our environment could alter AD risk through shifts in microbial metabolism.

These three studies have profoundly influenced my understanding of how our skin microbiome, our environment, and our immune system interact in many ways that impact our health. I really appreciated these works because they made significant progress in elucidating the underlying mechanisms that influence development of AD, identified the role of a relatively understudied skin commensal, and investigated the role of environmental exposures on these interactions. These works together have inspired me to view my own work on the role of skin commensals in immune signaling and *S. aureus* colonization resistance through the combined lenses of immunology, environment, and microbial metabolism.
